# Stress-dependent activation of myosin in the heart requires thin filament activation and thick filament mechanosensing

**DOI:** 10.1073/pnas.2023706118

**Published:** 2021-04-13

**Authors:** So-Jin Park-Holohan, Elisabetta Brunello, Thomas Kampourakis, Martin Rees, Malcolm Irving, Luca Fusi

**Affiliations:** ^a^Randall Centre for Cell and Molecular Biophysics, School of Basic and Medical Biosciences and British Heart Foundation Centre of Research Excellence, King’s College London, SE1 1UL London, United Kingdom

**Keywords:** heart muscle, myosin motor, muscle regulation, myosin-binding protein C

## Abstract

Myosin-based regulation in the heart muscle modulates the number of myosin motors available for interaction with calcium-regulated thin filaments, but the signaling pathways mediating the stronger contraction triggered by stretch between heartbeats or by phosphorylation of the myosin regulatory light chain (RLC) remain unclear. Here, we used RLC probes in demembranated cardiac trabeculae to investigate the molecular structural basis of these regulatory pathways. We show that in relaxed trabeculae at near-physiological temperature and filament lattice spacing, the RLC-lobe orientations are consistent with a subset of myosin motors being folded onto the filament surface in the interacting-heads motif seen in isolated filaments. The folded conformation of myosin is disrupted by cooling relaxed trabeculae, similar to the effect induced by maximal calcium activation. Stretch or increased RLC phosphorylation in the physiological range have almost no effect on RLC conformation at a calcium concentration corresponding to that between beats. These results indicate that in near-physiological conditions, the folded myosin motors are not directly switched on by RLC phosphorylation or by the titin-based passive tension at longer sarcomere lengths in the absence of thin filament activation. However, at the higher calcium concentrations that activate the thin filaments, stretch produces a delayed activation of folded myosin motors and force increase that is potentiated by RLC phosphorylation. We conclude that the increased contractility of the heart induced by RLC phosphorylation and stretch can be explained by a calcium-dependent interfilament signaling pathway involving both thin filament sensitization and thick filament mechanosensing.

## Significance

The efficiency of the heart as a pump depends on an autoregulatory mechanism, the Frank–Starling law of the heart, that potentiates the strength of contraction in response to an increase in ventricular filling. Disruption of this mechanism compromises the ability of the heart to pump blood, potentially leading to heart failure. We used fluorescent probes on myosin in heart muscle cells to investigate the molecular basis of the Frank–Starling mechanism. Our results show that the stronger contraction of heart muscle at longer lengths is due to a calcium-dependent interfilament signaling pathway that links stress sensing in the myosin-containing filaments with calcium activation of the actin-containing filaments. This pathway can potentially be targeted for treating heart failure.

The contraction of cardiac muscle is generated by reciprocal sliding of actin-containing thin filaments and myosin-containing thick filaments in the sarcomere driven by myosin motors. The interaction of the myosin motors with the overlapping thin filament is primarily controlled by calcium-induced structural changes in the thin filament linked to the intracellular calcium transient (1). Calcium ions released in the cytoplasm following an action potential bind to troponin, triggering the movement of tropomyosin around the filament, which uncovers actin sites to which the motors can bind and power contraction (2). However, some of the myosin motors may not be available for actin binding, as they are folded onto the thick filament surface in relaxing conditions (3). Thick filament–based regulatory mechanisms control the release of the myosin motors from the folded conformation and contribute to the regulation of contractility of striated muscle (3–6).

Electron microscopy (EM) studies on isolated thick filaments from vertebrate heart muscle showed that the myosin motors in the region of the filament that contains myosin-binding protein C (MyBP-C), the C-zone, are sequestered in helical tracks on the thick filament surface and are folded back onto their tails in an asymmetric conformation called the interacting-heads motif, or IHM (7, 8), originally identified in two-dimensional crystals of dephosphorylated smooth muscle myosin (9). The IHM has also been associated with a biochemical state of myosin with a very low adenosine triphosphate (ATP)-ase rate, called the “super-relaxed” state (10), which is considered to be an OFF state of myosin. A recent X-ray diffraction study of cardiac muscle (11) extended that concept and suggested that in diastole, the resting phase of the cardiac cycle, three distinct motor conformations coexist in the thick filament in roughly equal numbers: folded helical, folded nonhelical, and disordered. The folded helical motors are likely to correspond to the IHM conformation and are confined to the C-zone. All the folded motors would be unavailable for actin binding and therefore OFF, but the disordered motors would constitute a population of constitutively ON motors that are immediately available for actin binding upon activation of the thin filament.

Stress sensing in the thick filament can control the release of the myosin motors from the folded states and might be responsible for modulating the strength of contraction of cardiac muscle in response to changes in the afterload (i.e., the arterial pressure) (12). Moreover, the transitions between these motor conformations, together with the calcium-induced structural changes in the thin filament, control the speed of contraction and relaxation (11). According to this mechanosensing paradigm of thick filament regulation, the constitutively ON motors play a fundamental role in the activation of cardiac muscle, as the force generated by these motors immediately after the electrical stimulus triggers a positive mechanosensing feedback loop that controls the number of active motors and the dynamics of contraction. Destabilization of the folded conformations by mutations in myosin and other thick filament proteins can alter the equilibrium between these motor conformations, leading to a hypercontractile phenotype in some hypertrophic cardiomyopathies (HCM) (13, 14). Pharmacological therapies targeting thick filament proteins to treat HCM (15–17) have been aimed at reversing the destabilization of the folded states caused by these mutations.

The contractility of the heart is also controlled by phosphorylation of the myosin regulatory light chain (RLC) (18) and by β-adrenergic signaling pathways mediated by phosphorylation of MyBP-C in the thick filament (19) as well as troponin in the thin filament, which are also likely to alter the equilibrium between regulatory conformations of the motors. RLC phosphorylation is essential for the normal function of the heart. The pattern of contraction of the heart may depend on a spatial gradient of RLC phosphorylation across the ventricle wall (20), and a decrease in the level of RLC phosphorylation is associated with heart failure (21, 22). RLC phosphorylation potentiates the contractility of vertebrate and invertebrate striated muscle, an effect that is generally thought to be mediated by disrupting the folded helical conformation of the myosin motors on the thick filament and increasing the number of motors available for interaction with actin during contraction at a given [Ca^2+^] (23, 24). Disordering of the myosin motors on the thick filament by RLC phosphorylation has been shown in in vitro studies on isolated thick filaments (23) and is the main mechanism of thick filament activation in intact striated muscle of tarantula during contraction (25). However, the effect of RLC phosphorylation on the structure of the cardiac thick filament in diastole is unclear. More generally, the large changes in force and the speed of contraction and relaxation of cardiac muscle produced by β-adrenergic agonists are not associated with significant changes in the diastolic structure of the thick filament (26), so they are not simply mediated by increasing the number of ON motors in diastole. Similarly, length-dependent activation (LDA), the cellular correlate of the Frank–Starling law of the heart and a key autoregulatory mechanism that adjusts the cardiac output in response to different extents of diastolic filling (27, 28), seems not to be simply mediated by a stretch-induced change in the structure of the thick filament in diastole (11, 26). Conflicting results have been reported (29), however, and mathematical models have suggested that activation of myosin motors induced by the passive tension transmitted to the thick filament by titin might contribute to LDA in cardiac muscle (30, 31).

Here, we investigated the in situ conformation of the myosin motors and its dependence on temperature, RLC phosphorylation, [Ca^2+^], and sarcomere length (SL) in demembranated cardiac trabeculae from rat hearts using the polarized fluorescence from probes on the N- and C-lobes of the RLC (18, 32). We show that, at the low [Ca^2+^] values that maintain the relaxed state and at near-physiological temperature and lattice spacing, the RLC-lobe orientations are consistent with about one-third of the myosin motors being in the folded helical conformation corresponding to the IHM, likely stabilized by MyBP-C in the C-zone of the filament. At the low [Ca^2+^] values that maintain the relaxed state, the folded conformation of the myosin motors is disrupted by cooling but not by RLC phosphorylation or stretch. However, stretching cardiac muscle at higher [Ca^2+^] that partially activates the thin filament triggers a stress-dependent activation of the thick filament and a force increase that is potentiated by RLC phosphorylation. This increase in contractility, induced by RLC phosphorylation and stretch, can be explained by an interfilament signaling pathway that links the stress-dependent activation of the thick filament to the activation state of the thin filament.

## Results

### The Fraction of Folded Motors in Relaxed Trabeculae Is Modulated by Temperature and Interfilament Spacing.

**Fig. 1.**
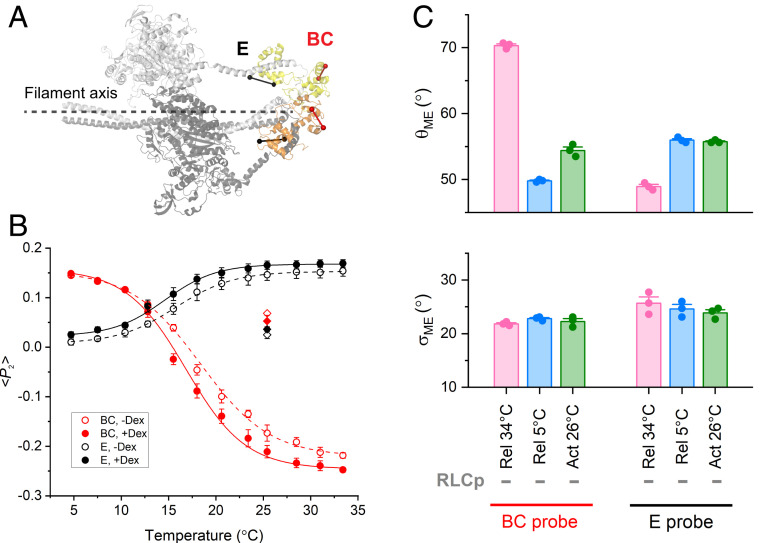
Temperature dependence of RLC N- and C-lobe orientation in relaxed trabeculae with unphosphorylated RLC. (*A*) Orientation of E (black) and BC (red) probes in the C- and N-lobe of RLC, respectively, in the blocked (orange) and free (yellow) head in the human β-cardiac myosin IHM (PDB 5TBY, essential light chains not shown for clarity), with respect to the filament axis (dashed line). (*B*) <*P*_2_> for E (black; mean ± SEM, *n* = 3) and BC (red; mean ± SEM, *n* = 3) probes in the absence (open circles) and in the presence (filled circles) of 3% Dextran T-500 in relaxed trabeculae at pCa 9 at 2.1 µm SL, fitted with Boltzmann curves (*SI Appendix*, Table S1 and *Materials and Methods*). Filled and open diamonds: values of <*P*_2_> (mean ± SEM, *n* = 3) at 26 °C and pCa 4.7 at the plateau of contraction in the presence (active isometric force per CSA [*T*_0_] = 84 ± 7 kPa; mean ± SEM, *n* = 6) and in the absence of Dextran (*T*_0_ = 78 ± 5 kPa; mean ± SEM, *n* = 6), respectively. Before activation, SL was set at 2.1 µm. (*C*) Mean angle (θ_ME_) and dispersion (σ_ME_) of the maximum entropy orientation distribution for E and BC probes in the presence of Dextran before RLC phosphorylation under relaxing conditions (pCa 9) at 34 °C (pink) and 5 °C (blue) and during active contraction (pCa 4.7) at 26 °C (green). Bars are mean ± SEM.

We used bifunctional rhodamine (BSR) probes on the E helix in the C-lobe (E probe) and cross-linking B- and C-helices in the N-lobe (BC probe) of the RLC of myosin to measure the in situ orientation of the two RLC lobes in demembranated cardiac trabeculae from rat hearts (Fig. 1*A*). A total of 43 ± 8% (mean ± SEM, *n* = 5) of the native RLC was replaced with labeled RLC (see *Materials and Methods*) with minimal effect on the isometric force during maximal calcium activation or on the rate of force redevelopment following unloaded shortening (*SI Appendix*, Fig. S1), indicating that the function of the myosin motors in situ was minimally affected by the RLC-exchange protocol.

The order parameters <*P*_2_>, <*P*_4_>, and <*P*_2d_> for the two BSR-RLCs, calculated from the polarized fluorescence intensities, were recorded in relaxed trabeculae (pCa = 9.0) in the temperature range 5 to 34 °C at 2.1 µm SL. <*P*_2_> is a measure of how parallel the probe dipole is to the filament axis and varies from +1 for parallel orientation to −0.5 for perpendicular orientation; <*P*_4_> is a higher-order harmonic term giving higher resolution angular information, while <*P*_2d_> quantifies rapid probe motion (33). Increasing the temperature of relaxed trabeculae toward the physiological value induced a sigmoidal increase in <*P*_2_> for the E probe (Fig. 1*B*, black open circles) with *T*_0.5_, the temperature at which the change in <*P*_2_> is half-maximal, about 16 °C (*SI Appendix*, Table S1). This result indicates that the E helix of RLC becomes more parallel to the thick filament axis at near-physiological temperature, as observed in relaxed skeletal muscle fibers (32). A larger change in <*P*_2_>, but in the opposite direction with *T*_0.5_ ∼18 °C, was observed for the BC probe (Fig. 1*B*, red open circles), suggesting that the dipole axis of the BC probe becomes more perpendicular to the thick filament at higher temperatures. Considering that the E and BC probes are parallel and perpendicular to the myosin lever arm, respectively (Fig. 1*A*), their orientation changes induced by increasing the temperature are consistent with tilting of the heavy chain axis in the light chain domain from a more perpendicular to a more parallel orientation with respect to the filament axis, as expected for a greater fraction of heads taking up the conformations seen in the IHM in isolated filaments.

Since demembranation of cardiac trabeculae induces an expansion of the myofilament lattice, we also investigated the effect of osmotic compression of the filament lattice by Dextran T-500 on the orientation of the RLC lobes. The addition of 3% [weight/volume (wt/vol)] Dextran to the relaxing solution caused a decrease in the cross-sectional area (CSA) of the trabecula by 25 ± 1% (mean ± SEM, *n* = 8), reversing the increase in myofilament lattice spacing associated with demembranation (34, 35). Dextran slightly increased <*P*_2_> for the E probe at each temperature and decreased that of the BC probe at temperatures above 10 °C (Fig. 1*B*, filled symbols). *T*_0.5_ for both probes was ∼1 to 2 °C lower in the presence of Dextran (*SI Appendix*, Table S1). Osmotic compression of the myofilament lattice in relaxed trabeculae slightly increased the fraction of myosin motors with their heavy chain helix in the light chain domain parallel to the filament axis (Fig. 1*A*), as observed in skeletal muscle fibers (32), but its effect was much smaller than that induced by increasing the temperature.

We made a quantitative comparison of the probe orientations inferred from these in situ polarized fluorescence measurements at near-physiological temperature (34 °C) and lattice spacing with those expected for the conformation of the helical folded myosin motors determined by three-dimensional EM of isolated filaments, the IHM (36) [Protein Data Bank (PDB) 5TBY; Fig. 1*A* and *SI Appendix*, Table S2], using a one-dimensional maximum entropy (ME) algorithm to calculate the broadest angular distribution consistent with the observed order parameters (37). The mean of the ME orientation distributions (θ_ME_) in these conditions is 70° for the BC probe in the N-lobe of the RLC (Fig. 1*C*, pink), close to the average of the values for the free and blocked heads in the IHM, 78° and 58°, respectively (*SI Appendix*, Table S2). However, θ_ME_ for the E probe in the C-lobe is 49° (Fig. 1*C*), much larger than the values for the free and blocked heads in the IHM, 23° and 16°, respectively (*SI Appendix*, Table S2). This result suggests that, in these near-physiological conditions, nearly all the myosin motors have their RLC N-lobe in a conformation similar to that in the IHM, but only a fraction of motors have their C-lobe in that conformation, as observed in skeletal muscle (32). Under the simplest assumption that motors with C-lobes not in the IHM conformation are disordered, that fraction is about one-third (*SI Appendix*).

Activation of cardiac trabeculae at maximal calcium concentration (pCa 4.7) at 26 °C, in both the presence and absence of Dextran, caused a decrease in <*P*_2_> for the E probe and an increase in that for the BC probe, with respect to their relaxed values, at forces of 80 to 90 kPa (Fig. 1*B*, diamonds). These changes are larger than those observed previously for the same probes in cardiac trabeculae using different experimental protocols, including activation at a lower temperature (∼20 °C) in the absence of Dextran (38), conditions that only partially preserve the folded orientation of the motors in relaxed trabeculae. In the conditions used here, the E probe became more perpendicular and the BC probe more parallel to the filament axis upon activation (Fig. 1*C*, green), as expected for a more perpendicular orientation of the heavy chain axis in the light chain domain of the active motors. Cooling relaxed trabeculae induced similar orientation changes in the E probe and larger changes in θ_ME_ of the BC probe (Fig. 1*C*, blue) but in the absence of force. Together, these results indicate that calcium activation and cooling in cardiac trabeculae in the absence of calcium induce similar orientation changes in the RLC, consistent with a large fraction of myosin motors leaving the folded conformation.

### The Effect of RLC Phosphorylation on the Fraction of Folded Motors in Relaxed Trabeculae.

**Fig. 2.**
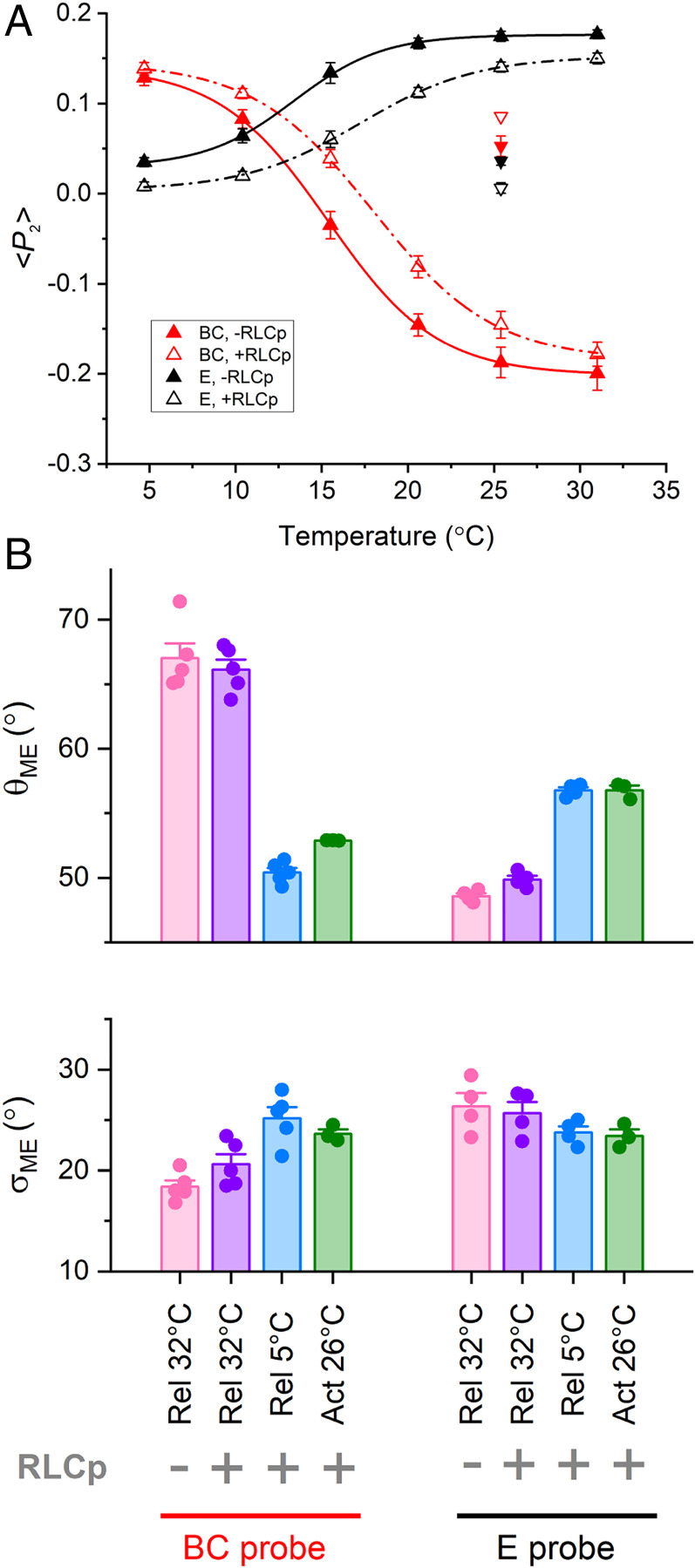
Temperature dependence of RLC N- and C-lobe orientation in relaxed trabeculae after RLC phosphorylation. (*A*) <*P*_2_> for E (black; mean ± SEM, *n* = 4) and BC (red; mean ± SEM, *n* = 5) probes before (filled triangles) and after (open triangles) RLC phosphorylation in relaxed trabeculae at pCa 9.0 at 2.1 µm SL in the presence of 3% Dextran, fitted with Boltzmann curves (*SI Appendix*, Table S1). Filled and open inverted triangles: values of <*P*_2_> at 26 °C and pCa 4.7 (mean ± SEM, *n* = 3) before (*T*_0_ = 84 ± 7 kPa; mean ± SEM, *n* = 6) and after RLC phosphorylation (*T*_0_ = 65 ± 6 kPa; mean ± SEM, *n* = 6), respectively. (*B*) Mean angle (θ_ME_) and dispersion (σ_ME_) of the maximum entropy orientation distribution for E and BC probes in the presence of Dextran under relaxing conditions at 32 °C before RLC phosphorylation (pink) and after RLC phosphorylation at 32 °C (violet) and 5 °C (blue) and during active contraction at 26 °C (green). Bars are mean ± SEM.

RLC phosphorylation was undetectable in freshly demembranated rat cardiac trabeculae in the conditions used in this study (*SI Appendix*, Fig. S2), as previously reported (39). We increased the level of RLC phosphorylation by incubating the RLC-exchanged trabecula in 2 µM smooth muscle myosin light chain kinase (smMLCK) and 2 µM calmodulin in activating solution (pCa 4.7) in the presence of 30 mM 2,3-butanedione monoxime (BDM) at 26 °C. After a 45 min incubation, both the native and labeled RLCs were phosphorylated to 0.47 ± 0.05 mol/mol (mean ± SEM, *n* = 5 trabeculae; *SI Appendix*, Fig. S2), close to the level in the healthy heart (21). In situ RLC phosphorylation increased *T*_0.5_ for <*P*_2_> in the presence of Dextran by 4 °C and 3 °C for the E and BC probes, respectively (Fig. 2*A*, open triangles; *SI Appendix*, Table S1). However, at a high temperature, RLC phosphorylation induced only small changes in <*P*_2_> for the E and BC probes toward their active values (Fig. 2*A*), consistent with only a small (∼1°) change in θ_ME_ for both probes (Fig. 2*B*). This result indicates that the relaxed orientations of both N- and C-lobes of RLC were only slightly altered by RLC phosphorylation at near-physiological temperature and lattice spacing, even though ∼50% of the RLCs were phosphorylated. Moreover, the changes in <*P*_2_> and θ_ME_ induced by either maximal activation at 26 °C or cooling in the absence of calcium were not significantly affected by RLC phosphorylation (Fig. 2), indicating that the orientation change of the RLC region of the myosin motors induced by these interventions is independent of RLC phosphorylation.

### RLC Orientation Is Insensitive to Stretch in Relaxed Trabeculae.

**Fig. 3.**
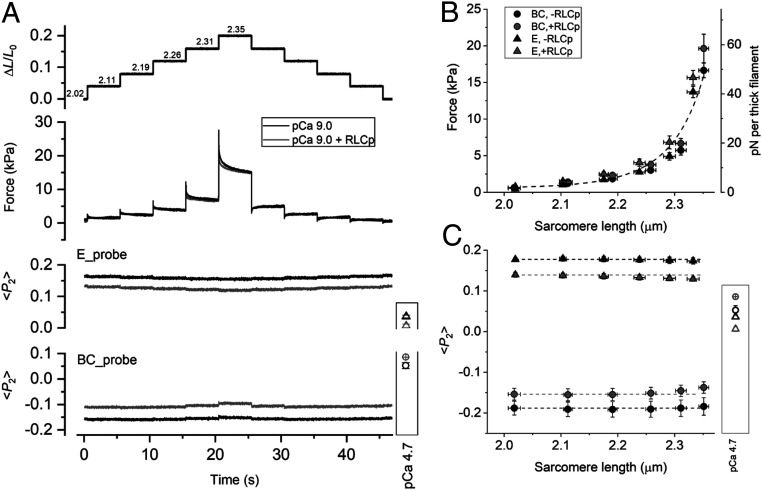
Effect of SL on RLC N- and C-lobe orientation in relaxed trabeculae. (*A*) Trabecular length change relative to the initial length (*L*_0_) in relaxed trabeculae at pCa 9.0 ([Ca^2+^] = 1 nM); the SL in micrometers shown above the trace was measured before the stretch staircase and calculated after each stretch from the relation in *SI Appendix*, Fig. S3. Passive force response and <*P*_2_> for E and BC probes were recorded in single trabeculae before (black) and after (gray) RLC phosphorylation. Values of <*P*_2_> during full calcium activation for the two probes (E, triangles; BC, circles) are shown in the box. Temperature 26 °C, 3% Dextran T-500. Dependence of passive force (*B*) and <*P*_2_> (*C*) on SL, recorded at the initial length and at 5 s after each stretch, before (black) and after (gray) RLC phosphorylation; triangles, E probe (mean ± SEM, *n* = 4); circles, BC probe (mean ± SEM, *n* = 5). Dashed line in *B* is the curve fitted to all data using the function F = exp(a + b × SL + c × SL^2^) (a = 87.5, b = −88.9 µm^−1^, c = 22.5 µm^−2^). Dashed lines in *C* denote relaxed <*P*_2_> values at short SL; active <*P*_2_> values are shown in the box (symbols as in *A*).

Next, we determined whether the conformation of the myosin motors in relaxed cardiac trabeculae (pCa 9.0) is sensitive to stretch, as observed in skeletal muscle (5) and expected in the simplest form of the mechanosensing hypothesis. We measured the changes in the orientation of the E and BC probes on the RLC in response to a staircase of stretches and releases at 26 °C in the presence of 3% (wt/vol) Dextran T-500 (Fig. 3*A*), conditions that maximize the fraction of folded motors and allow direct comparison with the effect of calcium activation on RLC-lobe orientation at the same temperature. SL was increased from 2.0 to 2.35 µm following a parabolic dependence on trabecular length (*SI Appendix*, Fig. S3). In this SL range, the passive tension induced by stretch is mainly accounted for by stretching the region of titin that connects the tip of the thick filament to the Z-line of the sarcomere (40). Passive tension showed viscoelastic behavior, increasing during each stretch and decaying during the 5 s interval before the next stretch, with a markedly different response to a ramp release at the same SL (Fig. 3*A*, black). The force measured 5 s after the stretch increased to ∼6 kPa, equivalent to ∼20 pN per thick filament at SL 2.3 µm, and then more steeply at longer SLs (Fig. 3*B*, black). However, the order parameters of the E and BC probes were almost unaffected by stretch over the whole range of SL (Fig. 3 *A* and *C*, black), particularly in comparison with those induced by cooling (Fig. 1) or maximal calcium activation (Fig. 3 *A* and *C*, box).

To investigate whether RLC phosphorylation increases the sensitivity of the myosin motors to stretch, we repeated the staircase protocol in the same trabeculae after in situ phosphorylation by smMLCK. Although RLC phosphorylation has a small effect on the conformation of the myosin motors as described above, it had no detectable effect on the passive force response (Fig. 3 *A* and *B*, gray). Moreover, the changes in probe orientation in response to stretch (Fig. 3 *A* and *C*, gray) were still much smaller than those induced by maximal calcium activation (Fig. 3*A*, box), indicating that even after RLC phosphorylation the orientations of both the N- and C-lobes of the RLC are almost insensitive to the stretch in relaxing conditions (pCa 9.0). Stretching relaxed trabeculae in the physiological SL range does not reduce the fraction of myosin motors in the folded state.

### Stretch Triggers Changes in RLC-Lobe Orientation at Higher [Ca^2+^] that Activates the Thin Filament.

**Fig. 4.**
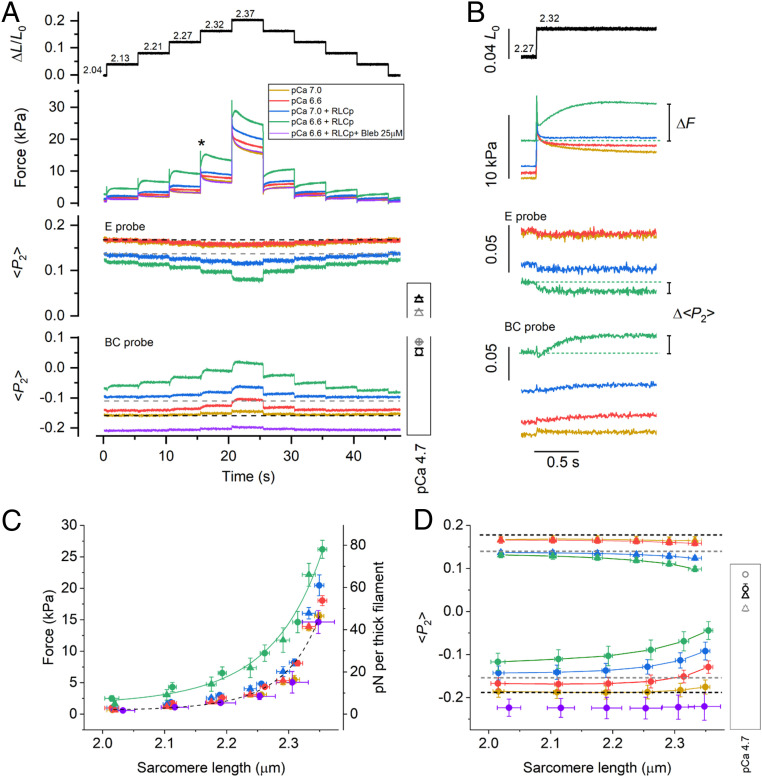
The effect of SL on RLC-lobe orientation in trabeculae activated at submaximal [Ca^2+^]. (*A*) Relative trabecular length change (Δ*L*/*L*_0_) and SL in µm calculated from the relation in *SI Appendix*, Fig. S3. Force and <*P*_2_> for E and BC probes during the stretch/release staircase recorded in single trabeculae at different [Ca^2+^] before and after RLC phosphorylation and in the presence of Blebbistatin 25 µM, as shown in the legend. Dashed lines: <*P*_2_> values in relaxing conditions (pCa 9) before (black) and after (gray) RLC phosphorylation. Values of <*P*_2_> for the two probes (E, triangles; BC, circles) during full calcium activation before (black) and after (gray) RLC phosphorylation are shown in the box (same as in Fig. 2*A*). Temperature, 26 °C; 3% Dextran T-500. (*B*) Traces at the time marked (asterisk) in *A* are shown on an expanded time scale; dashed lines indicate pre-stretch values. The dependence of force (*C*) and <*P*_2_> (*D*) on SL recorded before the stretch staircase and at 5 s after each stretch; triangles, E probe (mean ± SEM, *n* = 4); circles, BC probe (mean ± SEM, *n* = 4); same color code as in *A*. Data in the presence of 25 µM Blebbistatin (purple) are mean ± SD, *n* = 2. Dashed line in *C* is from Fig. 3*B*; solid line, curve fitted to green data points [F = exp(a + b × SL + c × SL^2^); a = 45.2, b = −47.1 µm^−1^, c = 12.4 µm^−2^]. Dashed lines in *D*: relaxed <*P*_2_> values at short SL before (black) and after (gray) RLC phosphorylation; active <*P*_2_> values are shown in the box (as in *A*).

Free [Ca^2+^] in cardiac muscle cells between beats, in diastole, is typically ∼100 nM (pCa 7.0) (41), much higher than the ca 1 nM (pCa 9.0) concentration in the relaxing solution used for the experiments described above, and can increase to 0.2 to 0.7 µM during systole (42). We next investigated whether the RLC-lobe orientation is sensitive to stretch in this physiological [Ca^2+^] range. At pCa 7.0 and at low RLC phosphorylation levels, trabecula remained fully relaxed and the orientation of the RLC lobes was close to that at pCa 9.0 (Fig. 4, yellow). Moreover, both the force and the <*P*_2_> responses to the staircase stretch (Fig. 4, yellow) were similar to those at pCa 9.0 (Fig. 3). At pCa 6.6, the trabecula was slightly activated (force ∼1 kPa, corresponding to ∼1 to 2% of the isometric force during maximal calcium activation) at short SL, and the force response to the stretches and the associated change in <*P*_2_> for the BC (but not the E) probe toward its active value became slightly larger (Fig. 4, red). These changes in probe orientation were fully reversed during the shortening staircase.

Physiological levels of RLC phosphorylation increase myofilament calcium sensitivity (18), and at pCa 7.0 and short SL, the trabecula was slightly activated (force ∼1 kPa). Under these conditions, the staircase stretch induced an increased response in force and in <*P*_2_> for both the E and BC probes but only at SLs >2.3 µm (Fig. 4, blue). At pCa 6.6 and short SL, the force increased (∼3 kPa) and <*P*_2_> for both probes showed larger changes (Fig. 4, green), indicating greater release of the N- and C-lobes of the RLC from their folded conformations. Under these conditions, each stretch in the staircase triggered a delayed increase in active force (Δ*F*; Fig. 4 *A* and *B*, green), a phenomenon known as stretch activation (SA) (43), accompanied by further <*P*_2_> changes for the two probes (Δ<*P*_2_>; Fig. 4*B*) toward their active values. SA caused an increase in the total force at each SL (Fig. 4*C*, green), and the steady values of <*P*_2_> for both the E and BC probes became closer to those associated with maximal calcium activation (Fig. 4*D*, green). Both the force and probe orientation changes were abolished in the presence of 25 μM Blebbistatin (Fig. 4, purple), which locks the myosin motors in their folded conformation (44).

**Fig. 5.**
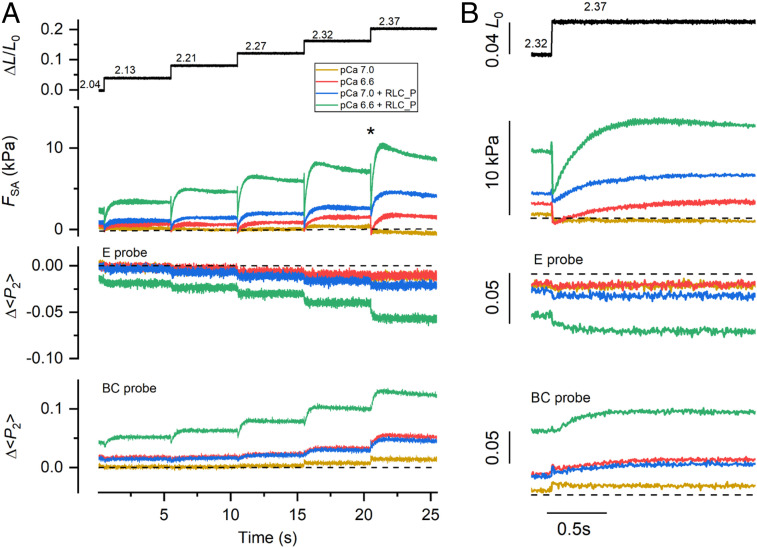
RLC phosphorylation enhances SA in partially activated cardiac trabeculae. (*A*) Relative trabecular length change and SL changes in micrometers (calculated from *SI Appendix*, Fig. S3), *F*_SA_, and <*P*_2_> changes (Δ<*P*_2_>) for E and BC probes with respect to their relaxed values (pCa 9.0) during the stretch staircase. (*B*) Traces at the time marked (asterisk) in *A* shown on an expanded time scale. Dashed lines mark the zero.

We calculated the active force component of SA (*F*_SA_) at each pCa by subtracting the passive force at pCa 9.0 (Fig. 3) from the total force response (Fig. 4). At pCa 7.0, *F*_SA_ was close to zero at each SL (Fig. 5, yellow). At pCa 6.6, *F*_SA_ decreased in the first ∼20 ms after the stretch and then increased exponentially with a rate constant of ∼5 s^−1^ with a larger amplitude at longer SL (Fig. 5, red). The increase in *F*_SA_ was accompanied by an increase in <*P*_2_> (Δ<*P*_2_>) for the BC probe, which also had a rate constant of ∼5 s^−1^ (Fig. 5*B*). RLC phosphorylation enhanced SA at pCa 7.0 and 6.6 by increasing *F*_SA_ and Δ<*P*_2_> for both BC and E probes at each SL (Fig. 5, blue and green). These results indicate that the RLC remains largely insensitive to stretch at calcium concentrations that do not activate the thin filament. However, at calcium concentrations and RLC phosphorylation levels that partially activate the thin filament, the stretch triggered SA and the release of RLC N- and C-lobes from their folded orientation. Moreover, the force and the changes in RLC orientation in the steady state of the SA response were similar to those induced by imposing the same stretch in relaxing conditions and then activating the trabecula by increasing [Ca^2+^], the sequence that would occur in the physiological LDA response (*SI Appendix*, Fig. S4). This result suggests that SA and LDA involve the same mechanism of stretch-induced activation of folded myosin motors. Consistent with that interpretation, the effects of stretch and RLC phosphorylation on the calcium sensitivity of both force and <*P*_2_> for the RLC probes in the SA protocol used here (*SI Appendix*, Fig. S5) are similar to those observed in steady-state calcium titrations at different SLs (18).

### The Dependence of RLC-Lobe Orientation on Filament Stress.

**Fig. 6.**
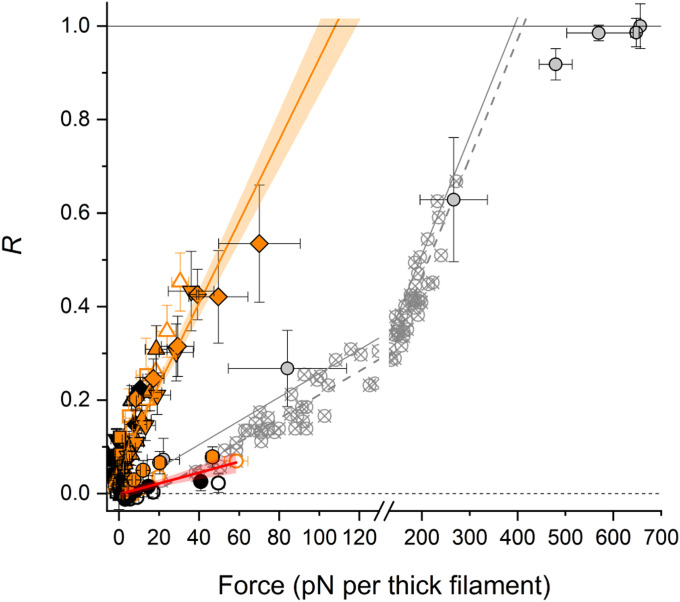
The dependence of the RLC-lobe orientation on filament stress. Dependence of the relative change in probe orientation (*R*) on passive (circles, pCa 9.0) and active filament stress in the presence of calcium (squares, pCa 7.00; triangles, pCa 6.60; inverted triangles, pCa 6.48; and diamonds, pCa 6.34) in cardiac trabeculae before (black) and after (orange) RLC phosphorylation. Filled symbols, E probe (mean ± SEM, *n* = 4); open symbols, BC probe (mean ± SEM, *n* = 5). Short dashed line marks the zero. Red line, linear regression on the data at pCa 9 with 95% confidence bands [slope = (1.2 ± 0.2) × 10^−3^ pN^−1^; intercept = 0.002 ± 0.005]. Orange line: linear regression on the data at pCa < 9.0 with 95% confidence bands [slope = (8.7 ± 0.4) × 10^−3^ pN^−1^; intercept = 0.059 ± 0.006]. In gray, dependence of *R* on passive force in the presence of 25 µM Blebbistatin (crossed circles) and on active force (circles; mean ± SEM, *n* = 5) in the pCa range 9 to 4.7 for the skeletal RLC E probe in muscle fibers, calculated from figure 5 in Fusi et al. (5). Dashed and solid gray lines, linear regressions on passive [slope = (2.5 ± 0.1) × 10^−3^ pN^−1^; intercept = −0.042 ± 0.009] and active [slope = (2.5 ± 0.2) × 10^−3^ pN^−1^; intercept = 0.003 ± 0.01] data, respectively.

To further characterize the relationship between thick filament stress and the changes in orientation of the RLC lobes in response to the stretch, we described the level of activation of the two RLC lobes in terms of the parameter *R* (= Δ<*P*_2_>/Δ<*P*_2_>_pCa4.7_), which expresses the change in <*P*_2_> for a given probe relative to the change measured during maximal calcium activation. In relaxed trabeculae (pCa 9.0), the increase in passive tension in the physiological SL range induced a small increase in *R* for E and BC probes (Fig. 6, filled and open circles, respectively), described by a shallow linear regression (Fig. 6, red line), indicating that the orientation of both the RLC N- and C-lobe is relatively insensitive to passive tension transmitted to the thick filament by titin. The slope of this relationship is similar to that observed for the E helix probe on the skeletal RLC in skeletal muscle fibers (5) (Fig. 6, crossed circles and dashed line), indicating a similar dependence of the RLC C-lobe orientation on passive filament stress in the two muscle types.

In the pCa range 7.00 to 6.34, before RLC phosphorylation, *R* for both the E and BC probe (Fig. 6 and *SI Appendix*, Fig. S6, black symbols) measured immediately before each stretch in the staircase protocol increased slightly with the active filament stress (*F*_SA_) up to a maximum of ∼0.2. After RLC phosphorylation, *R* for both probes increased up to ∼0.5 in the same pCa range, with a similar linear dependence on the stretch-activated force (Fig. 6 and *SI Appendix*, Fig. S6, orange line) and with a steepness that is approximately eight times larger than that in relaxing conditions and approximately four times larger than that for the RLC E helix in actively contracting skeletal muscle at different [Ca^2+^] (Fig. 6, gray circles and solid line). This result indicates that the orientation of the RLC lobes in cardiac muscle is more sensitive to active than passive filament stress and that RLC phosphorylation enhances the stress-dependent orientation changes in both lobes without altering the relationship between orientation and stress.

## Discussion

The results presented establish the conditions under which the myosin motors on the thick filament in demembranated cardiac trabeculae recover the physiological conformation typical of the diastolic phase of the cardiac contractile cycle. First, we showed that the regulatory state of the myosin motors in the relaxed muscle cell is strongly dependent on temperature (Fig. 1). Increasing the temperature toward the physiological value triggers a conformational change in the nucleotide binding site of the myosin head associated with the ATP hydrolysis by myosin and the formation of its closed conformation that promotes the helical packing of the myosin motors on the thick filament (45, 46). Our data extend previous studies on the effect of temperature on the conformation of the myosin motor to its RLC region by showing that, at higher temperature, a larger fraction of the myosin motors have their RLC region in the folded helical IHM conformation inferred from EM of isolated cardiac thick filaments (36). Conversely, cooling relaxed trabecula, which favors the ATP-bound or open state of myosin, disrupts the folded conformation of the myosin motors in the absence of filament force, and the RLC region is tilted away from the filament axis, similar to the effect of maximal calcium activation. The structural change induced by cooling involves a change in mean orientation rather than an increase in orientational dispersion (Figs. 1 and 2) in marked contrast with previous studies in which the cooling-induced transition was interpreted as the loss of an ordered conformation (46).

**Fig. 7.**
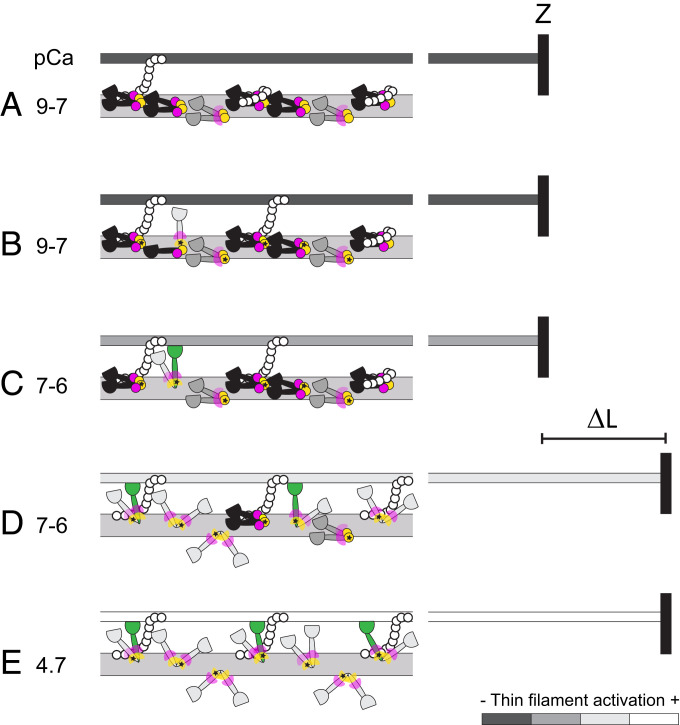
A working hypothesis for the changes in the conformation of myosin motors in the C-zone of the thick filament induced by RLC phosphorylation and stretch. (*A*) In the relaxed cardiac trabecula (pCa 9 to 7), folded helical myosin motors (black) have RLC N- and C-lobes in IHM orientations (magenta and yellow circles, respectively). Folded nonhelical (gray) motors have only the N-lobe in IHM orientations and the C-lobe in conformational equilibrium (magenta wedges). MyBP-C (white circles) in the C-zone may link to actin or tether folded helical motors. The thin filament (dark gray) is fully switched off. Z, Z-disk. (*B*) Physiological levels of RLC phosphorylation (star) release only a few motors from the folded conformation (light gray) and favor the formation of MyBP-C links with actin. (*C*) The thin filament is partially activated by calcium (pCa > 7) and by MyBP-C links, allowing myosin motors to bind to actin and generate force (green). (*D*) The stretch (ΔL) triggers the stress-dependent activation of the myosin motors and may promote the formation of additional MyBP-C links that further increase the activation of the thin filament, allowing more force-generating motors to bind to actin. (*E*) Fully ON state of thin and thick filaments at maximal calcium concentration (pCa 4.7).

The folded conformation of myosin that is populated at near-physiological temperature is further stabilized in situ by compressing the filament lattice to restore the physiological distance between thin and thick filaments, an effect that might be mediated by an interfilament signaling pathway in which MyBP-C links the thick and thin filaments (47). However, the present results reveal a different involvement of the N- and C-lobes of the RLC in the folded conformation. At a near-physiological temperature and lattice spacing, most of the motors have their RLC N-lobes in IHM-like conformations (Fig. 7*A*, yellow spheres). These IHM-like N-lobe conformations are probably stabilized by docking onto the filament surface by interactions between the RLC–RLC regions of the blocked and free head of individual myosins (36) and by intermolecular interactions between adjacent myosin molecules (7), as suggested by previous studies using multiple probes on the RLC N-lobe (38). Mutations in the N-lobe associated with a severe HCM phenotype may alter the regulation of the myosin motors in cardiac muscle by destabilizing these interactions (48). In contrast, the order parameters for the C-lobe are consistent with only one-third of the motors on the thick filament being in an IHM-like conformation in relaxed cardiac trabeculae at a near-physiological temperature and lattice spacing (Fig. 7*A*, black myosin motors). A qualitatively similar conclusion was reached in previous studies with multiple probes on the RLC in relaxed skeletal muscle fibers (32) and cardiac trabeculae (38), suggesting that in both skeletal and cardiac muscle, the RLC C-lobes of most myosin motors are not in the IHM-like conformation. The IHM conformation has only been observed by EM in the C-zone of the cardiac thick filament, which contains about half of the motors, and within the C-zone in only two out of every three myosin crowns (7) (Fig. 7*A*). The estimate of one-third of the RLC C-lobes being in the IHM conformation therefore matches the fraction of C-zone motors assigned to that conformation by EM studies.

The orientations of both the N- and C-lobes are not altered by increasing [Ca^2+^] from the relaxed (pCa 9.0) to the diastolic value (pCa 7.0). It follows that the above conclusions about the orientations of the N- and C-lobes of the RLC in relaxed trabeculae can be extended to the ionic conditions in diastole, at least in demembranated trabeculae. A further extension of that conclusion to intact trabeculae in diastole is supported by recent X-ray diffraction studies on cardiac trabeculae from rat hearts showing that the thick filament structure in demembranated trabeculae in relaxing solution in the presence of 3% Dextran T-500 at 27 to 37 °C is similar to that in intact quiescent trabeculae (34), and that in intact trabeculae in diastole half of the folded motors are helically ordered and confined to the C-zone of the filament, whereas the other half are not helical and are mainly localized in the D-zone (11). The corollary would be that the motors identified by X-ray diffraction as folded helical have both RLC lobes in IHM-conformations, whereas the disordered and the folded nonhelical motors are likely to have a mixed population of RLC C-lobe orientations but an ordered IHM-like N-lobe (Fig. 7*A*).

To better mimic the physiological condition of cardiac muscle cells, we phosphorylated, in situ, the myosin RLC to a level typical of the healthy heart (∼40 to 50%) (21). We found that at near-physiological temperature and lattice spacing, physiological levels of RLC phosphorylation do not significantly alter the diastolic conformation of the myosin motors (Fig. 7*B*), in marked contrast with the changes induced by maximal calcium activation (Fig. 7*E*). This was an unexpected result, since in vitro studies on isolated skeletal thick filaments (23) and recent in situ studies on skeletal muscles of tarantula (25) suggested that RLC phosphorylation largely disorders the structure of the thick filament in relaxing conditions. Moreover, previous studies with the BC probe in cardiac trabeculae using different experimental protocols at ∼20 °C in the absence of Dextran showed that RLC phosphorylation of about 50% led to a change in probe orientation almost as large as that associated with calcium activation (18). The difference is likely to be due to the stabilization of the folded conformation of the myosin motors at higher temperature in the presence of Dextran (Fig. 2). In those more physiological conditions, phosphorylation of about half of the RLCs has a very small effect on the number of folded motors in diastole.

To test the hypothesis that the thick filament in cardiac muscle acts as a stress sensor, as it does in skeletal muscle (4, 5), we studied the effect of passive tension on the conformation of the myosin motors. Under relaxing conditions (pCa 9 to 7), the present results show that the passive stress generated by stretching titin in the physiological SL range from 2.0 to 2.3 µm has almost no effect on the folded conformation of the myosin motors. The relationship between motor conformation and passive filament stress is almost the same in relaxed skeletal and heart muscle (5) (Fig. 6), but the motor conformation is affected so little by stretch in heart muscle because the passive tension remains below 20 pN per thick filament despite increasing exponentially with SL in the physiological range. This passive filament stress is too small to activate the cardiac thick filaments via the mechanosensing mechanism. Our results therefore argue strongly against the hypothesis that the Frank–Starling law of the heart and its cellular correlate, LDA, is mediated by a regulatory structural change in the thick filament triggered by passive stress (29).

The present results show that neither LDA nor RLC phosphorylation are mediated by a change in structure of the thick filament in near-physiological diastolic conditions, though both are associated with increased active force generation in the presence of calcium associated with an increase in the calcium sensitivity of the thin filament (18, 27). We conclude that the potentiation of cardiac contractility induced by RLC phosphorylation and LDA is likely mediated by a calcium-dependent interfilament signaling pathway. The present results also show that the delayed active force response (SA) triggered by stretching cardiac muscle cells in the systolic [Ca^2+^] range (0.2 to 0.7 µM) (42) is accompanied by stress-dependent regulatory structural changes in the thick filament that are enhanced by RLC phosphorylation. SA controls force generation during oscillatory work of the indirect flight muscle of insects (49) and has been proposed to modulate systolic force in the myocardial layers that are stretched during the early phases of systole (43, 50). Mechanical studies on insect flight muscle have suggested that SA might be mediated by regulatory structural changes in the thin filament induced by stretching actin-attached myosin motors (51), but the molecular mechanism underlying SA in the heart remained unclear. The present experiments showed that the steady-state SA response of cardiac muscle is similar to that induced by a stretch that precedes myofilament activation (*SI Appendix*, Fig. S4), the LDA protocol that is related to the Frank–Starling law of the heart (28). This result indicates that SA and LDA, and the associated change in myofilament calcium sensitivity, might share the underlying mechanisms related to the release of myosin motors from the filament surface. The similarity of the steady-state force component of the SA and LDA responses was already implied by the observation that the force at a given [Ca^2+^] and SL depends only on the final SL and is independent of the sequence of length changes by which it is reached (52, 53). SA and LDA may be mediated by an interfilament signaling pathway involving MyBP-C (11, 54, 55), which could link the stress-dependent activation of the thick filament to the activation state of the thin filament. By sharing part of this pathway, RLC phosphorylation and stretch could have additive effects on the activation of the thin filament. RLC phosphorylation is likely to destabilize the interaction of the N-terminal domain of MyBP-C with the RLC of the folded motors favoring the formation of MyBP-C links with actin that sensitize the thin filament to calcium (54, 55) (Fig. 7 *A* and *B*).

The mechanism by which increased SL signals to the thin filament to increase its calcium sensitivity remains unclear. One widely discussed hypothesis proposes that the decrease in interfilament spacing mediates the effect of stretch, and this could facilitate the formation of additional MyBP-C links between the filaments (Fig. 7 *C* and *D*). However, the role of a change in interfilament spacing in LDA remains controversial (35, 56). Another possibility is that stress-dependent activation of the thick filament might directly destabilize the interaction of the N terminus of MyBP-C with myosin, allowing more thin-filament MyBP-C links to form.

Calcium binding to the M-domain of MyBP-C might also directly control the conformation of the N terminus of MyBP-C and increase its binding affinity for the thin filament (57, 58). The structure and function of the MyBP-C N terminus is also regulated by phosphorylation (57, 59), supporting the hypothesis of a signaling role for MyBP-C in LDA. However, alternative mechanisms of interfilament communication cannot be excluded. Interactions between titin and actin, troponin, or tropomyosin might alter the activation level of the thin filament at longer SL (28, 60).

Our results describe a fundamental aspect of thick filament mechanosensing in cardiac muscle. In contrast with the corresponding mechanosensing mechanism in skeletal muscle (61), according to which the thick filaments are activated equally by passive or active stress (5), the folded motors on the cardiac thick filament have an eightfold higher sensitivity to active than passive stress. This additional graded release of folded myosin motors would consequently be tuned to the physiological load applied on the cardiac thick filament during systole (12). Indeed, extrapolation of the linear stress dependence of the folded motors (Fig. 6) suggests that the full activation of the thick filament would be reached at a filament force of ∼110 pN, close to the systolic filament force developed during the cardiac twitch of ∼130 pN at 26 °C in rat cardiac trabeculae (11), which is five times smaller than the isometric filament force in skeletal muscle at the same temperature (∼650 pN in rabbit psoas muscle fibers) (5).

The higher gain of the mechanosensing mechanism in the cardiac thick filament proposed above might also explain the higher cooperativity of its calcium-dependent activation measured with RLC probes (18), compared to that in skeletal muscle (5), and might also be involved in the observed coupling of the rates of force generation and relaxation in cardiac muscle (62). The higher gain of the mechanosensing transition in the thick filaments in heart muscle would facilitate sequestration of the myosin motors detached from actin during the initial phase of relaxation onto the filament surface, thereby controlling the dynamics of force relaxation (11). Moreover, the high gain of the positive mechanosensing feedback in the cardiac thick filament implies that small perturbations of the conformational equilibrium between folded and constitutively ON motors in diastole, such as those caused by HCM-causing mutations in myosin or MyBP-C (13) or myosin-targeting drugs (15–17), might significantly affect the stress-dependent release of folded motors in systole and their inhibition during diastole, consequently affecting the strength of contraction and the rate of relaxation of cardiac muscle, respectively. Because the Frank–Starling mechanism might be depressed or absent in end-stage failing human myocardium due to dilated cardiomyopathy (63), and because a blunted LDA response is a common pathogenic mechanism underlying cardiac dysfunction in HCM (64), the thick filament–based mechanisms and the interfilament signaling pathway at the basis of LDA described here represent primary targets for the development of new pharmacological approaches to treat heart failure in those diseases.

## Materials and Methods

The protocols for the preparation of demembranated cardiac trabeculae, BSR-labeled cardiac regulatory light chains (cRLCs), smMLCK and calmodulin, and the composition of the physiological buffers are described in *SI Appendix*, *Materials and Methods*.

### Experimental Protocol.

The trabeculae were mounted in a temperature-controlled multidrop apparatus in relaxing solution between the levers of a strain gauge force transducer and a loudspeaker linear motor using aluminum clips attached to the ends of the trabecula (65). Before each experiment, the ends of the trabeculae were fixed with shellac dissolved in ethanol at 2 °C. The clip-to-clip trabecular length (*L*_0_), CSA, and the SL were measured under the microscope with an immersion objective (40×) in the absence of Dextran T-500 and after stretching to just above slack length. In the trabeculae used for these experiments, *L*_0_ = 960 ± 235 µm, and CSA = 11,388 ± 5,161 µm^2^ (*n* = 18). The active and passive forces measured in the trabeculae were normalized by the CSA in the absence of Dextran and expressed in kPa. The force per thick filament was calculated from the force in kPa, taking into account a fractional occupancy of the myofibrils of 0.6 and the filament lattice spacing, *d*_1,0_ = 39.5 nm, measured in demembranated trabeculae in the absence of Dextran (34). The calculated density of thick filaments in the demembranated trabecula is 0.56 × 10^15^ m^−2^. To test the mechanical properties of the trabecula, SL was set to 2.1 µm in relaxing conditions, and the trabecula was activated at maximal calcium (pCa 4.7) at 26 °C using a temperature-jump (T-jump) protocol; the trabecula was initially incubated in preactivating solution at 2 °C for 5 min and then transferred to activating solution at 2 °C for ∼7 s to reach a steady force, then to the same solution at 26 °C, and finally back to relaxing solution (*SI Appendix*, Fig. S1). A ramp release (amplitude ∼0.07 *L*_0_ in 2 ms) was applied at the plateau of contraction after the T-jump to measure the rate of force redevelopment at the end of the shortening. The isometric force before the ramp was 109 ± 23 kPa (*n* = 18), corresponding to a force per thick filament of ∼320 pN. The RLC probes were exchanged in the trabecula using the same protocol for the RLC exchange into skeletal muscle fibers (32); the trabecula was washed in rigor solution at 2 °C for 3′ three times and in RLC-exchange buffer at 2 °C for 3′, and then it was incubated in RLC-exchange buffer containing ∼20 µM of RLC probe at 19 °C for 40′ and washed in relaxing solution at 10 °C for 5′. Finally, the trabecula was incubated in relaxing solution at 10 °C containing ∼20 µM of wild-type human cardiac troponin C for 30′ and then washed in relaxing solutions at 30 °C to remove any nonspecifically bound probe. After the exchange, 43 ± 8% (mean ± SEM, n= 5 trabeculae) of the native RLC was replaced by the RLC probe, as estimated from densitometric analysis of the blots (*SI Appendix*, Fig. S2), and the force during T-jump activation at 26 °C was 88 ± 2% (mean ± SEM; *n* = 12) of the value recorded before RLC exchange. To determine the SL dependence on trabecular length, we measured SL under the microscope after increasing the trabecular length in steps of ∼0.04 *L*_0_ up to a maximum change of ∼0.2 *L*_0_ under relaxing conditions (*SI Appendix*, Fig. S3). The order parameters of the RLC probes were measured in relaxing conditions (pCa 9.0) in the temperature range 5 to 35 °C in the absence and in the presence of 3% (wt/vol) Dextran. The measurements were carried out at the highest temperature first; then, after cooling the trabecula decreasing the temperature in steps of ∼6 °C, and finally, after warming it up again interpolating the previous temperature values. In the trabeculae used to characterize the effect of RLC phosphorylation, the order parameters were measured in the presence of Dextran changing the temperature in steps of ∼6 °C. The order parameters recorded at a high temperature before and after cooling were similar, indicating that the changes in orientation induced by cooling were completely reverted by warming up the relaxed trabecula. The dependence of the orientation of the RLC probes on SL was investigated at 26 °C in the presence of Dextran by increasing the trabecular length in relaxing conditions, using a series of five stretches with amplitude 0.04 *L*_0_ complete in 5 ms applied every 5 s, followed by a staircase of releases with the same amplitude, while the order parameters were continuously sampled at 200 Hz. The stretch-release staircase was repeated at pCa 7.0, 6.60, 6.48, 6.34, and, finally, 9.0 again. The passive force responses at pCa 9.0 recorded at the beginning and at the end of the protocol were similar, indicating that the passive force response of the cardiac trabecula was reproducible and not significantly affected by the stretches at higher [Ca^2+^]. To phosphorylate in situ the RLC, the trabecula was incubated first in relaxing buffer at 26 °C in the presence of 30 mM BDM for 10′ and then in activating buffer at 26 °C in the presence of 30 mM BDM, 2 µM calmodulin, and 2 µM smMLCK for 45′, and, finally, it was washed in relaxing solution. After this protocol, the RLC was phosphorylated to 0.47 ± 0.05 mol/mol (mean ± SEM, *n* = 5 trabeculae; *SI Appendix*, Fig. S2). The temperature dependence of the order parameters of the RLC probes in relaxing conditions and their SL dependence in the range 9.00 to 6.34 pCa were characterized after RLC phosphorylation using the same protocols described above. Finally, the trabecula was activated with T-jump at maximal calcium concentration at 26 °C to measure the isometric force after RLC phosphorylation, and then it was dismounted from the setup for quantification of the RLC exchange and phosphorylation using Phostag sodium dodecyl sulphate–polyacrylamide gel electrophoresis (SDS-PAGE) and subsequent Western blot analysis of cRLC (*SI Appendix*, Fig. S2), as previously described (18). The analysis of the fluorescence polarization data is described in *SI Appendix*.

### Statistical Analysis.

All data were analyzed using dedicated programs written in LabVIEW (National Instruments), Microsoft Excel, and Origin 2019 (OriginLab Corp.) software. Error bars on mean data points are ±SEM. For mean data in the figures, the number of independent observations (*n*) corresponds to the number of cardiac trabeculae used and is reported in the legends. Only cardiac trabeculae that developed an isometric force of ∼100 kPa in the first test contraction at maximal calcium (26 °C) were used for the experimental protocol described in *Materials and Methods*.

## Supplementary Material

Supplementary File

## Data Availability

All study data are included in the article and/or *SI Appendix*.
