# Supplementary material for: Stress-dependent activation of myosin in the heart requires thin filament activation and thick filament mechanosensing
Source: Proc Natl Acad Sci U S A. 2021 Apr 13;118(16):e2023706118. doi: 10.1073/pnas.2023706118 (PMC8072254; doi:10.1073/pnas.2023706118)
Supplement: Supplementary File [file pnas.2023706118.sapp.pdf]

PNAS
www.pnas.org

Supplementary Information for
Stress-dependent activation of myosin in the heart requires thin filament activation and thick filament mechano-sensing.

So-Jin Park-Holohan, Elisabetta Brunello, Thomas Kampourakis, Martin Rees, Malcolm Irving, Luca Fusi*.

Randall Centre for Cell and Molecular Biophysics, School of Basic and Medical Biosciences and BHF Centre of Research Excellence, King's College London, London SE1 1UL, UK.

Corresponding author: Luca Fusi
luca.fusi@kcl.ac.uk

This PDF file includes:

Supplementary Methods
Figures S1 to S6
Tables S1 to S2
SI References

Supplementary Methods

Preparation of BSR-cRLC.

Mutants of the human ventricular cRLC (UniProtKB entry: MLRV_HUMAN; P10916) with pairs of cysteines introduced at positions 54 and 63 on helices B and C respectively in the N-lobe of RLC, and at positions 97 and 110 on helix E in the C-lobe of RLC were obtained by site-directed mutagenesis, as previously described (1). The mutants were expressed in E. coli BL21(DE3) cells (Stratagene) as N-terminal fusion proteins with a Histidine tag (His-tag) and TEV protease site from a pET6a vector. The N-terminal tag-sequence was removed by digestion with TEV protease leaving three additional amino acids (glycine-serine-serine) at the N-terminus. Each of the cRLC double-cysteine mutants was labelled with BSR-I2 (Invitrogen, B-10621) and purified by ion-exchange chromatography on a MonoS column to 95% homogeneity. The protein was concentrated to over 1 mg/ml and stored in 25 µl aliquots at -80ºC for further use.

Preparation smMLCK.

The DNA encoding residues 1445-1903 of smooth muscle MLCK (MYLK, NM_053025.3), encompassing the kinase and following immunoglobulin domain, was cloned into a modified pENTRY vector containing an N-terminal His-tag and then recombined into baculovirus DNA with the BaculoDirectTM Baculovirus Expression system (Invitrogen). The resulting virus was used to infect SF9 (*Spodoptera Frugiperda*) cells, with protein expressed for 3 days at 28°C with shaking at 100rpm. smMLCK was purified by nickel affinity chromatography on a HisTrap column (GE Heatlhcare), followed by size exclusion chromatography on a Superdex 75 column (GE Healthcare) equilibrated in 20mM HEPES pH 7.5, 100mM NaCl, 1mM DTT. The protein was concentrated to 294µM, flash frozen and stored at -80°C.

Preparation of Calmodulin.

The DNA sequence encoding Calmodulin (CALM3, NM_001329922.1, isoform CRA_e) was cloned into a modified pET vector and expressed in E. coli BL21 (DE3) cells. Calmodulin was purified by hydrophobic interaction chromatography on a Phenyl-Sepherose column (GE Healthcare), and fractions containing pure calmodulin were pooled, buffer exchanged into 10mM Tris pH 8.0, 0.1mM EDTA, concentrated to 1mM, flash frozen and stored at -80°C.

Preparation of cardiac trabeculae and physiological buffers.

Rattus norvegicus, strain Wistar Han (male, 6-8 weeks old) were supplied by Charles River Laboratories. The rats were euthanized by cervical dislocation after sedation with isoflurane in compliance with the UK Home Office Schedule 1 and European Union regulation (directive 2010/63), followed by a confirmation method. The heart was rapidly excised and cannulated via the ascending aorta and retrogradely perfused with a modified Krebs-Henseleit buffer (119 mM NaCl, 5mM KCl, 0.5 mM $CaCl_2$, 1.2 mM NaH_2PO_4, 1.2 mM $MgSO_4$, 25mM $NaHCO_3$, 10mM glucose, 25 mM BDM) equilibrated with carbogen (95% O_2, 5% CO_2). Single unbranched trabeculae were dissected from the right ventricle under a stereomicroscope and were demembranated for 20 min on ice in relaxing solution in the presence of BDM (25 mM) and Triton X-100 1% (v/v), then stored at -20°C in storage solution (6 mM Imidazole, 70 mM Potassium Propionate (KPr), 8 mM $MgAc_2$, 5 mM EGTA, 7 mM Na_2ATP, 1 mM NaN_3, 50% glycerol) for up to 5 days. Relaxing solution contained: 25 mM Imidazole, 45 mM KPr, 6.89 mM $MgAc_2$, 10 mM EGTA, 5.56 mM Na_2ATP, 20 mM Na_2-creatine phosphate (CP), (pCa= -log [Ca^{2+}]= 9). Pre-activating solution contained: 25 mM Imidazole, 46 mM KPr, 6.48 mM $MgAc_2$, 0.1 mM EGTA, 9.9 mM HDTA, 5.6 mM Na_2ATP, 20 mM Na_2CP (pCa 9). Activating solution contained: 25 mM Imidazole, 46 mM KPr, 6.39 mM $MgAc_2$, 10mM Ca-EGTA, 5.65 mM Na_2ATP, 20 mM Na_2CP (pCa 4.7). Rigor solution contained: 25 mM Imidazole, 134 mM KPr, 1.5 mM $MgAc_2$, 10 mM EGTA (pCa 9). Solutions at submaximal [Ca^{2+}] were prepared by mixing relaxing and activating solutions: solutions with pCa 7.0, 6.60, 6.48 and 6.34 were obtained with 37.5, 62.5, 68.75 and

75% (v/v) of activating solution, respectively. In all the solutions free [Mg^{2+}]= 1.0 mM, ionic strength=180 mM and pH 7.1 at 25°C. The RLC-exchange buffer contained: 20 mM EDTA, 50 mM KPr, 10 mM Potassium Phosphate buffer, pH=7.1. On the day of the experiment the osmotic agent dextran T500 (3% w/v) was added to all experimental solutions, except rigor and RLC-exchange buffers, to reduce the inter-filament spacing to a value similar to that of intact trabeculae (2). Just before the experiment Protease inhibitor cocktail P8340 (Sigma) and 2 mM DTT were added to all the solutions.

Analysis of fluorescence polarisation data and estimate of the fraction of RLC N- and C-lobes in IHM orientations.

The order parameters $<P_2>$, $<P_4>$ and $<P_{2d}>$ of the RLC probes were calculated from the polarised fluorescent intensities recorded from the trabecula (3), using a software written in LabView. $<P_{2d}>$ for the two probes, quantifying the rapid probe motion, were 0.663 ± 0.024 (mean ± SEM, N=4) and 0.760 ± 0.021 (mean ± SEM, N=5) for E and BC probe respectively, in relaxing conditions (pCa 9.0) and in the presence of 3% Dextran at 32°C. $<P_{2d}>$ for both probes did not change significantly with decreasing the temperature to 5°C, in the absence of Dextran or after RLC phosphorylation, indicating that changes in the $<P_2>$ and $<P_4>$ measured in the different conditions were not affected by changes in the rapid probe motion. The temperature dependence of $<P_2>$ in relaxed trabeculae (Figs. 1,2) was fitted with a Boltzmann curve:

$$\boldsymbol{y} = \frac{<P_2>_{LT} - <P_2>_{HT}}{1+e^{(T-T_{0.5})/dT}} + <\boldsymbol{P_2}>_{HT}$$

in which $<P_2>_{LT}$ and $<P_2>_{HT}$ are the values of $<P_2>$ at low and high temperatures respectively, $T_{0.5}$ is the half-maximal transition temperature and dT is the slope of the sigmoid; the fit parameters of the curves in Figs.1,2 are shown in Table S1.

The mean angle θ_{ME} of the probe dipole with respect to the fibre axis and the mean dispersion σ_{ME} at 32°C were calculated from the Maximum Entropy (ME) distribution of θ_{ME}, as described in Julien et al. (4).

The *in situ* orientational distribution of each RLC lobe is not uniquely defined by the two order parameters measured using a single probe on each lobe and it is possible that the real distribution is more complex, i.e. that there are multiple populations of myosin motors with their N- and C-lobes in different orientations as inferred from a previous study of relaxed trabeculae which used four different probes on each lobe (1). However, the results from multiple probes would still not define a unique orientation distribution and would depend on the assumption that the tertiary structure of the lobe *in situ* was the same as that determined by protein crystallography or NMR. Given those limitations, the more productive approach is to compare the order parameters from a single probe on each lobe with those expected from a reference structure like the IHM. This approach still relies on the simpler assumptions that the probe dipole is parallel to its attachment points on the RLC and that the orientation of the lobe is not altered by the introduction of the probe. Therefore, to estimate the fraction of RLC N- and C-lobes in IHM orientations we calculated the expected values of $<P_2>$ and $<P_4>$ for the BC and E probes in the IHM corresponding to the probe angles θ for the free and blocked heads (Table S2), and compared them with the values determined by polarised fluorescence. θ was defined as the angle of the vector joining the β-carbons of the two RLC residues cross-linked by the probe with respect to the filament axis in the PDB 5TBY (5) (Fig. 1A). The expected values of $<P_2>$ and $<P_4>$ (Table S2) were calculated assuming a Gaussian orientation distribution with peak angle θ and standard deviation σ=15°, as described in Julien et al. (4). Because $<P_2>$ and $<P_4>$ have a linear dependence on the fraction of probes in a particular orientation (3), the order parameters of the E

and BC probes in the IHM are equal to the average of the order parameters of each probe in the free- and blocked-head. Thus, $<P_2>_{IHM}$ = 0.754, $<P_4>_{IHM}$ = 0.414 for the E probe, $<P_2>_{IHM}$ = -0.192, $<P_4>_{IHM}$ = -0.035 for the BC probe. The expected values for the BC probe are similar to those measured at 32°C in the presence of Dextran ($<P_2>$= -0.20 ± 0.02, $<P_4>$= 0.05 ± 0.04 mean ± SEM, N=5), indicating that most of the BC probes are in IHM-like orientation. In contrast, the expected values for the E probe are much larger than the experimental ones ($<P_2>$= 0.18 ± 0.01, $<P_4>$= 0.13 ± 0.05 mean ± SEM, N=4), suggesting that a large fraction of E probes are in orientations different from those in the IHM. Assuming that f_{IHM} is the fraction of E probes on the thick filament with IHM-like orientation and 1- f_{IHM} is, for simplicity, isotropic ($<P_2>$, $<P_4>$ = 0), then the order parameters of the orientation distribution are calculated as $<P_2> = f_{IHM}\cdot<P_2>_{IHM}$ and $<P_4> = f_{IHM}\cdot<P_4>_{IHM}$. With f_{IHM} = 0.3, $<P_2>$ and $<P_4>$ are 0.22 and 0.12 respectively, close to the experimental values.

Supplementary Figures

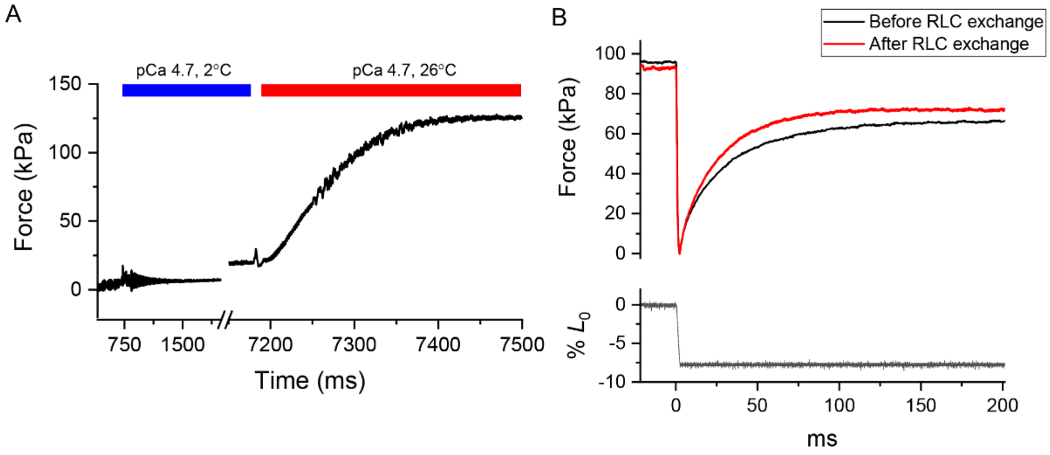

Fig. S1. Temperature-jump activation (T-jump) in cardiac trabeculae. (*A*) Force generation in a trabecula during about 7s activation at pCa 4.7 at 2°C and after transferring (at 7200 ms) the trabecula to activating solution at 26°C. (*B*) Force response of a trabecula to a ramp release (amplitude ~7% of the initial trabecular length L_0) applied at the plateau of contraction after the T-jump at 26°C, before (black) and after (red) RLC exchange.

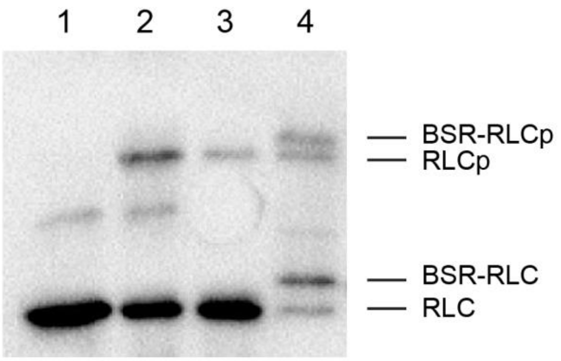

Fig. S2. *In situ* phosphorylation of RLC by myosin light chain kinase (MLCK). Phostag™ SDS-PAGE and subsequent Western-blot analysis of cRLC in single demembranated trabeculae. *Lane 1* (from the left): analysis of one trabecula after the demembranation protocol (see Methods). No significant RLC-phosphorylation was observed. *Lane 2-3*: analysis of two trabeculae after the *in situ* RLC phosphorylation protocol. The native RLC is partially phosphorylated (RLCp). *Lane 4*: analysis of one trabecula exchanged with the RLC-BC probe (BSR-RLC), phosphorylated while mounted on the experimental setup and dismounted at the end of the fluorescence polarisation experiment. Both the native and labelled RLC are phosphorylated. The fraction of RLC exchanged and phosphorylated in each trabecula was estimated by densitometric analysis using the software ImageJ.

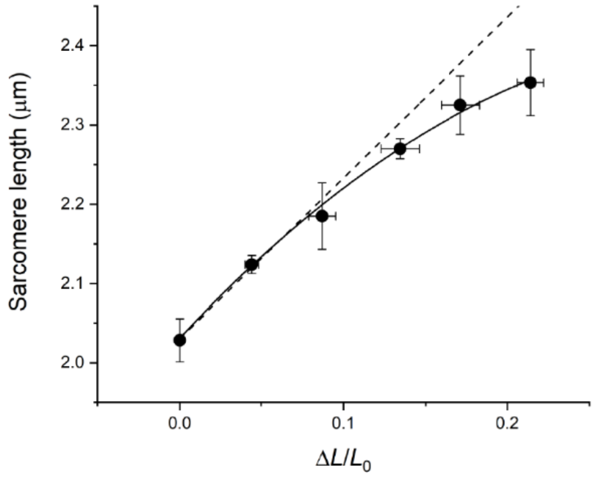

Fig. S3. Dependence of sarcomere length on the length change applied to a relaxed trabecula. The initial length of the relaxed trabecula (L_0) was adjusted to set the sarcomere length to ~2.0 μm (SL_0). The length of trabecula was increased in five steps of amplitude ~0.04 L_0 and the sarcomere length was measured after each stretch (mean ± SD, N = 6 trabeculae). Solid line is the parabola fitted to the data ($SL = a+b \cdot \Delta L+c \cdot \Delta L^2$; a = 2.031 ± 0.006 μm, b = 2.22 ± 0.14 μm, c = -3.25 ± 0.69 μm). Dashed line denotes the linear relation between sarcomere length and the trabecular length change ($SL = SL_0 \cdot (1+\Delta L/L_0)$). The deviation from linearity of this relationship at SL>2.2 μm is due to stretch of the end-compliance of the trabeculae.

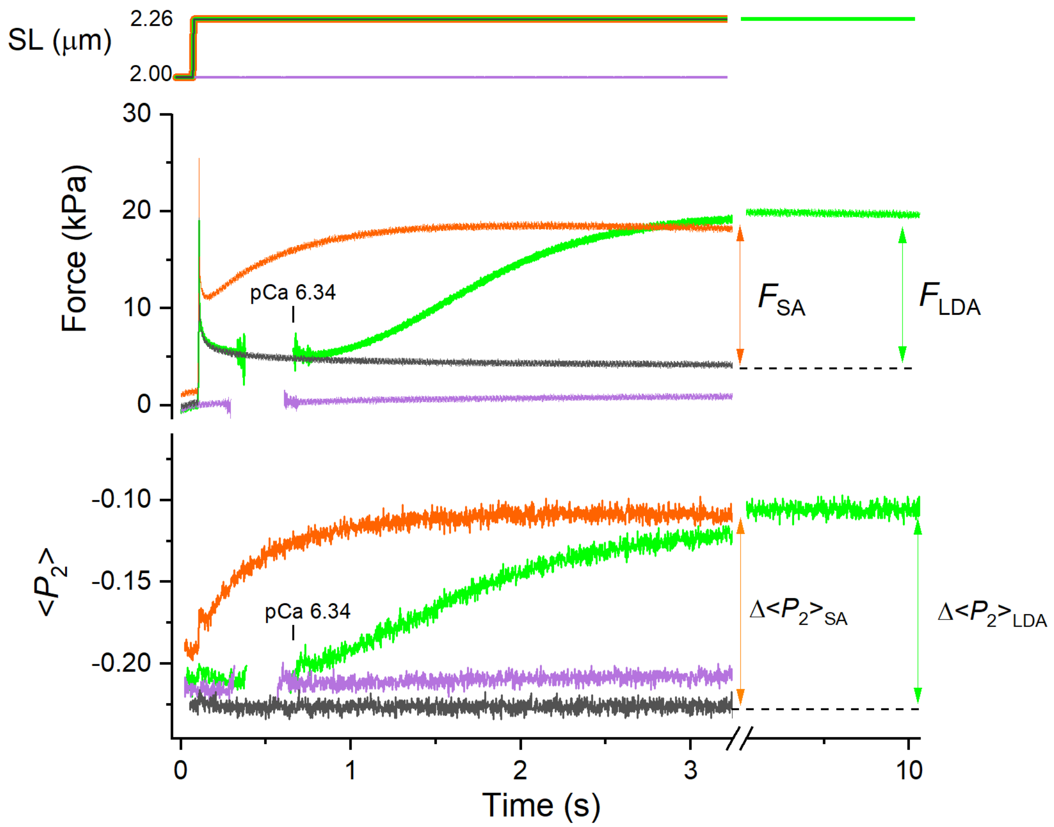

Fig. S4. Response of a cardiac trabecula to a stretch applied before or after calcium activation. Time courses of force and <P_2> for the RLC BC probe in response to the (i) stretch (complete in 5 ms) of the relaxed trabecula (pCa 9.0; black traces), (ii) stretch of the relaxed trabecula followed by activation at pCa 6.34 (green traces, LDA protocol; data points during the solution exchange are omitted and the vertical black mark indicates the time at which the trabecula enters the activating solution at ~0.7s from the stretch), (iii) stretch after activation of the trabecula at pCa 6.34 (orange traces; SA protocol), and (iv) activation at pCa 6.34 in the absence of stretch (purple traces; the vertical mark indicates the time at which the trabecula enters the activating solution).The stretch in relaxing conditions does not induce a change in probe orientation. The LDA and SA protocols induce similar active force potentiation (*F*) and change in <P_2> with respect to the relaxed value (Δ<P_2>) at the steady state. The slower force and <P_2> response in the LDA protocol are due to the slow calcium diffusion inside the cardiac trabecula during activation. Temperature, 26°C; 3% (w/v) Dextran T-500.

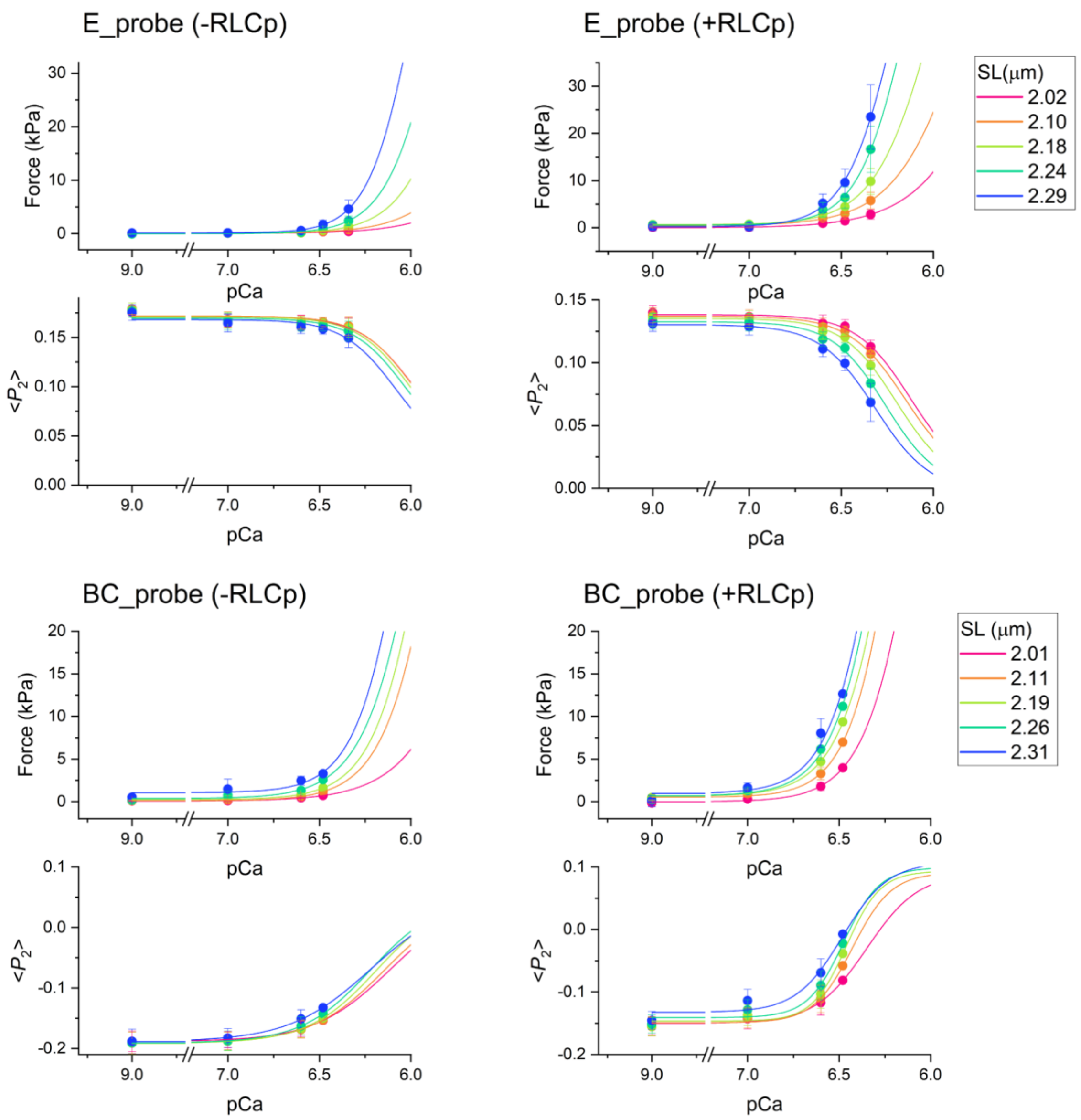

Fig. S5. Calcium dependence of force and <P_2> for RLC BC and E probes at different sarcomere lengths before and after RLC phosphorylation. Active force data and <P_2> for RLC BC and E probes at different sarcomere length (SL) and RLC phosphorylation levels, as shown in Fig. 4, are plotted against calcium concentration. Hill curves fitted to the data are defined as $y=B_{pCa9}+A*(1/(1+(10\string^((x-pCa_{50})*n))))$. The maximal force at pCa 4.7 was fixed at 90 kPa, close to that measured during T-jump activation (Fig. 1-2 and Fig. S1) and consistent with the sarcomere length dependence of active force in cardiac trabeculae (6). The maximal <P_2> change at pCa 4.7 was fixed to the <P_2> change from relaxing to activating conditions at 26°C as shown in Fig. 2A. Temperature, 26°C; 3% (w/v) Dextran T-500.

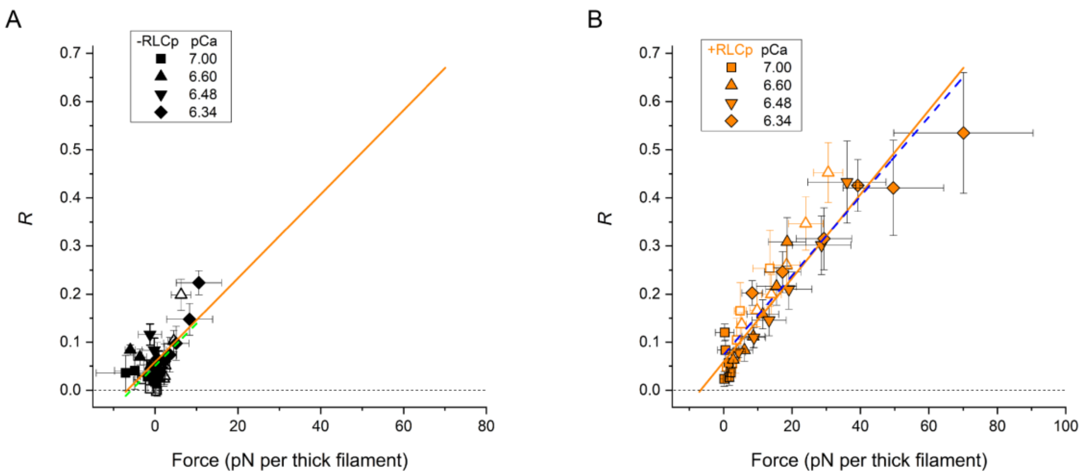

Fig. S6. The dependence of the RLC-lobe orientation on active filament stress in cardiac trabeculae. Data from Fig. 6 showing the dependence of the relative change in probe orientation (*R*) on active filament stress in the pCa range 7.00-6.34, as shown in the key, in cardiac trabeculae before (A) and after (B) RLC phosphorylation. Filled symbols, E probe (mean ± SEM, N = 4); open symbols, BC probe (mean ± SEM, N = 5). Orange line, linear regression on data in A and B, as shown in Fig. 6 (slope = $(8.7 \pm 0.4)\cdot 10^{-3}$ pN^{-1}; intercept = 0.059 ± 0.006). (A) Green dashed line, linear regressions on the data before RLC phosphorylation (slope = $(8.8 \pm 2.0)\cdot 10^{-3}$ pN^{-1}; intercept = 0.051 ± 0.007). (B) Blue dashed line, linear regression on the data after RLC phosphorylation (slope = $(8.3 \pm 0.6)\cdot 10^{-3}$ pN^{-1}; intercept = 0.07 ± 0.01).

BC probe	-Dex	+Dex	-RLCp	+RLCp
$<P_2>_{LT}$	0.154 ± 0.005	0.158 ± 0.004	0.141 ± 0.002	0.145 ± 0.0029
$<P_2>_{HT}$	-0.221 ± 0.007	-0.246 ± 0.005	-0.201 ± 0.001	-0.185 ± 0.005
$T_{0.5}$	18.37 ± 0.27	16.83 ± 0.28	15.37 ± 0.065	18.00 ± 0.18
dT	3.67 ± 0.22	3.15 ± 0.22	3.165 ± 0.063	3.50 ± 0.15
**E probe**	**-Dex**	**+Dex**	**-RLCp**	**+RLCp**
$<P_2>_{LT}$	0.007 ± 0.001	0.022± 0.004	0.030 ± 0.002	0.004 ± 0.001
$<P_2>_{HT}$	0.153 ± 0.001	0.168 ± 0.002	0.176 ± 0.001	0.152 ± 0.001
$T_{0.5}$	15.73 ± 0.11	14.58 ± 0.32	13.39 ± 0.18	17.22 ± 0.13
dT	3.03 ± 0.12	2.71 ± 0.25	2.57 ± 0.14	3.35 ± 0.13

Table S1. Fitted parameters for the temperature dependence of $<P_2>$ for E and BC probes. Parameters of Boltzmann curves (see SI Methods) fitted to the relaxed $<P_2>$ data for E and BC probes at different temperatures in the absence or presence of 3% Dextran T-500 (Fig. 1B), and before and after RLC phosphorylation (Fig. 2A). $T_{0.5}$ is the temperature at which the change in $<P_2>$ is half-maximal.

	E probe			BC probe		
	**θ**	**$\langle P_2 \rangle$**	**$\langle P_4 \rangle$**	**θ**	**$\langle P_2 \rangle$**	**$\langle P_4 \rangle$**
**Free head**	23°	0.704	0.319	78°	-0.347	0.103
**Blocked head**	16°	0.805	0.510	58°	-0.037	-0.173

Table S2. Probe orientations in the cardiac IHM. Angles θ for the E and BC probe dipoles with respect to the filament axis in the RLC of the free and blocked head in the human β-cardiac myosin IHM fitted into mass distributions of myosin motors determined by 3D reconstruction of the C-zone of the cardiac thick filament (PDB 5TBY) (Fig. 1A). $\langle P_2 \rangle$ and $\langle P_4 \rangle$ were calculated for each orientation assuming a Gaussian distribution of angular orientations with mean angle θ and dispersion σ=15° (see Supplementary Methods).

SI References

1. T. Kampourakis, Y. B. Sun, M. Irving, Orientation of the N- and C-terminal lobes of the Myosin regulatory light chain in cardiac muscle. *Biophys. J.* **108**, 304-314 (2015).
2. J. G. Ovejero *et al.*, The OFF-to-ON transition of thick filaments in isolated trabeculae from rat heart induced by cooling *Biophys. J.* **116**, 263A (2019).
3. R. E. Dale *et al.*, Model-independent analysis of the orientation of fluorescent probes with restricted mobility in muscle fibers. *Biophys. J.* **76**, 1606-1618 (1999).
4. O. Julien *et al.*, Toward protein structure in situ: comparison of two bifunctional rhodamine adducts of troponin C. *Biophys. J.* **93**, 1008-1020 (2007).
5. L. Alamo *et al.*, Effects of myosin variants on interacting-heads motif explain distinct hypertrophic and dilated cardiomyopathy phenotypes. *Elife* **6** (2017).
6. J. C. Kentish, H. E. ter Keurs, L. Ricciardi, J. J. Bucx, M. I. Noble, Comparison between the sarcomere length-force relations of intact and skinned trabeculae from rat right ventricle. Influence of calcium concentrations on these relations. *Circ Res* **58**, 755-768 (1986).
